# Alcançando as Metas de Colesterol LDL: A Ciência da Implementação Poderá nos Salvar?

**DOI:** 10.36660/abc.20250516

**Published:** 2025-09-10

**Authors:** Fernando Yue Cesena

**Affiliations:** 1 Instituto Dante Pazzanese de Cardiologia Seção de Hipertensão e Nefrologia São Paulo SP Brasil Instituto Dante Pazzanese de Cardiologia – Seção de Hipertensão e Nefrologia, São Paulo, SP – Brasil

**Keywords:** Doenças Cardiovasculares, Prevenção Secundária, Anticolesterolemiantes, Ciência da Implementação

A doença cardiovascular aterosclerótica (DCVA) é a principal causa de morte em todo o mundo,^1 , 2^ e indivíduos que já sofreram um evento de DCVA são os que têm maior risco de um novo evento.^3^ Alcançar níveis baixos de colesterol LDL (LDL-c) é uma estratégia comprovada e segura para prevenir eventos subsequentes. As diretrizes atuais recomendam níveis de LDL-c abaixo de 50 mg/dL ou 55 mg/dL na prevenção secundária.^3 , 4^ Os tratamentos disponíveis, incluindo estatinas, ezetimiba, agentes anti-PCSK9 e ácido bempedoico, são suficientes para que a maioria dos pacientes atinja as metas de LDL-c. No entanto, o fracasso generalizado em atingir essas metas tem sido amplamente relatado em todo o mundo.^5 – 10^

Esta edição dos Arquivos Brasileiros de Cardiologia apresenta um estudo que investigou as práticas de prescrição de 29 cardiologistas brasileiros para o manejo do LDL-c em prevenção secundária, estimando o número de pacientes potencialmente elegíveis para terapias além de estatinas e ezetimiba. Médicos que trabalham no Sistema Único de Saúde (SUS) ou Sistema de Saúde Suplementar (SSS) responderam a um questionário sobre suas práticas clínicas. Os autores estimaram que, entre os pacientes cujos cardiologistas visavam metas de LDL-c (92% do total), 79% no SUS e 86% no SSS atingiriam os níveis desejados de LDL-c usando apenas estatinas e ezetimiba. Ao estimar o número total de indivíduos em prevenção secundária no Brasil em 2024, os autores concluíram que mais de um milhão de indivíduos no SUS e ~150.000 no SSS seriam candidatos potenciais à terapia hipolipemiante além de estatinas e ezetimiba.^11^ Esses resultados fornecem suporte convincente para melhorar o acesso a medicamentos modernos para redução de lipídios, como agentes anti-PCSK9.

Esses achados também devem ser considerados no contexto mais amplo de adesão inadequada às diretrizes e alcance das metas de LDL-c abaixo do ideal observado na prática clínica. No Brasil, apesar de uma taxa de prescrição de estatinas superior a 80-85% na alta hospitalar ser frequentemente relatada após síndromes coronarianas agudas,^1^ o cenário para a doença cardíaca isquêmica crônica parece ser muito diferente. No registro da *NEtwork to control AtheroThrombosis* (NEAT), envolvendo 2.003 pacientes com doença arterial coronariana ou periférica, 55% dos que estavam em terapia com estatinas não utilizavam estatinas de alta intensidade. Além disso, a mediana do nível de LDL-c foi de 79 mg/dL, bem acima da meta recomendada pelas diretrizes, com apenas 8,6% dos participantes atingindo níveis de LDL-c abaixo de 55 mg/dL.^12^ Ainda mais alarmantes são os dados do programa Estratégia de Saúde da Família, que mostraram que, entre mais de 35.000 adultos com histórico de infarto do miocárdio ou acidente vascular cerebral, apenas 6,7% estavam em terapia com estatinas, e apenas 0,6% usavam estatinas de alta potência.^13^

As razões subjacentes ao controle inadequado do LDL-c são multifatoriais e amplamente documentadas.^6^ Fatores relacionados aos médicos incluem conhecimento insuficiente das diretrizes, ceticismo em relação às recomendações, receio quanto a potenciais efeitos adversos, inércia terapêutica e limitações de tempo para o atendimento ideal ao paciente. O presente estudo relata vários achados relevantes: apenas 57% dos cardiologistas do SSS concordaram com a meta da diretriz brasileira de LDL-c < 50 mg/dL; 50% dos cardiologistas do SUS relataram que poderiam usar sinvastatina como prescrição inicial; 17% não intensificariam a terapia quando as estatinas não atingem as metas de LDL-c; e 25% não concordaram com a prescrição de estatinas para todos os pacientes em prevenção secundária.^11^ Embora as razões por trás dessas práticas não tenham sido exploradas, os resultados indicam que há muito espaço para melhorias na transferência de diretrizes baseadas em evidências para a prática clínica de rotina.

Da perspectiva do paciente, a adesão aos tratamentos médicos é frequentemente comprometida por fatores como custos dos medicamentos, efeitos colaterais e conscientização limitada sobre o risco cardiovascular e os benefícios do tratamento.^5 , 14^ Em particular, deve-se priorizar a melhoria do acesso dos pacientes de maior risco a estatinas de alta intensidade, ezetimiba e terapias anti-PCSK9.

Essas observações ressaltam a importância da ciência da implementação, que é o estudo de métodos e estratégias para identificar e abordar barreiras à adoção de diretrizes e facilitar a transferência de recomendações baseadas em evidências para a prática clínica de rotina. Tem sido proposto um modelo de intervenção multinível direcionado a pacientes, profissionais de saúde, instituições clínicas e políticas de saúde.^15^ Em um relatório especial do *American College of Cardiology* e da *American Heart Association* , uma revisão sistemática identificou duas estratégias de intervenção particularmente eficazes para melhorar o processo de atendimento e os resultados clínicos: auditoria do desempenho clínico com recomendações de feedback e realização de visitas educacionais aos profissionais de saúde.^15^

Em conclusão, evidências substanciais indicam que existem oportunidades significativas para melhorar o controle do LDL-c entre pacientes com DCVA no Brasil e no mundo, reduzindo, em última análise, a carga de doenças cardiovasculares. Nossa incapacidade coletiva de transformar evidências clínicas em ações permanece evidente. Incentivar e apoiar a pesquisa de implementação é essencial para identificar e promover as estratégias mais eficazes para integrar as recomendações das diretrizes à prática clínica.
