# Supplementary material for: Alcançando as Metas de Colesterol LDL: A Ciência da Implementação Poderá nos Salvar?
Source: Arq Bras Cardiol. 2025 Sep 10;122(7):e20250516. [Article in Portuguese] doi: 10.36660/abc.20250516 (PMC12695075; doi:10.36660/abc.20250516)
Supplement: Translation

# Translation: Achieving LDL-Cholesterol Goals: Can Implementation Science Save Us?

**DOI:** 10.36660/abc.20250516i

**Authors:** Fernando Yue Cesena

**Affiliations:** 1 São Paulo SP Brazil Instituto Dante Pazzanese de Cardiologia – Seção de Hipertensão e Nefrologia, São Paulo, SP – Brazil

**Keywords:** Cardiovascular Diseases, Secondary Prevention, Anticholesteremic Agents, Implementation Science

Atherosclerotic cardiovascular disease (ASCVD) is the leading cause of death worldwide,^1 , 2^and individuals who have already experienced an ASCVD event are at the highest risk of a new event.^3^Achieving low levels of LDL-cholesterol (LDL-c) is a proven and safe strategy for preventing subsequent events. Current guidelines recommend LDL-c levels below 50 mg/dL or 55 mg/dL in secondary prevention.^3 , 4^Available therapies, including statins, ezetimibe, anti-PCSK9 agents, and bempedoic acid, are sufficient for most patients to achieve LDL-c targets. Nevertheless, the widespread failure to attain these goals has been extensively reported globally.^5 – 10^

This issue of *Arquivos Brasileiros de Cardiologia* presents a study that investigated the prescription practices of 29 Brazilian cardiologists for LDL-c management in secondary prevention, estimating the number of patients potentially eligible for therapies beyond statins and ezetimibe. Physicians working in the Public Health System (PHS) or Supplementary Health System (SHS) completed a questionnaire regarding their clinical practices. The authors estimated that, among patients whose cardiologists aimed for LDL-c targets (92% of the total), 79% in the PHS and 86% in the SHS would achieve the desired LDL-c levels using statins and ezetimibe alone. By estimating the total number of individuals in secondary prevention in Brazil in 2024, the authors concluded that over one million individuals in the PHS and ~150.000 in the SHS would be potential candidates to lipid-lowering therapy beyond statins and ezetimibe.^11^These results provide compelling support for improving access to modern lipid-lowering medications, such as anti-PCSK9 agents.

These findings should also be considered within the broader context of suboptimal guideline adherence and LDL-c target attainment observed in clinical practice. In Brazil, despite a statin prescription rate exceeding 80–85% at hospital discharge is often reported following acute coronary syndromes,^1^the scenario for chronic ischemic heart disease seems to be very different. In the NEtwork to control AtheroThrombosis (NEAT) registry, involving 2,003 patients with coronary or peripheral artery disease, 55% of those on statin therapy were not using high-intensity statins. Furthermore, the median LDL-c level was 79 mg/dL, which is well above the guideline-recommended target, with only 8.6% of participants achieving LDL-c levels below 55 mg/dL.^12^Even more alarming are data from the Family Health Strategy ( *Estratégia de Saúde da Família* ) program, which showed that, among more than 35,000 adults with a history of myocardial infarction or stroke, merely 6.7% were on statin therapy, and only 0.6% were using high-intensity statins.^13^

The reasons underlying inadequate LDL-c control are multifactorial and extensively documented.^6^Physician-related factors include insufficient knowledge of guidelines, skepticism about recommendations, apprehension about potential adverse effects, therapeutic inertia, and time constraints for optimal patient care. The current study reports several relevant findings: only 57% of SHS cardiologists agreed with the Brazilian guideline target of LDL-c <50 mg/dL; 50% of PHS cardiologists reported they could use simvastatin as the initial prescription; 17% would not escalate therapy when statins fail to achieve LDL-c targets; and 25% did not agree with prescribing statins to all patients in secondary prevention.^11^Although the reasons behind these practices were not explored, the results indicate substantial room for improvement in translating evidence-based guidelines into routine clinical practice.

From the patient's perspective, adherence to medical treatments is often compromised by factors such as medication costs, side effects, and limited awareness of cardiovascular risk and treatment benefits.^5 , 14^In particular, improving access for the highest-risk patients to high-intensity statins, ezetimibe, and anti-PCSK9 therapies should be prioritized.

These observations underscore the importance of implementation science, which is the study of methods and strategies to identify and address barriers to guideline adoption and facilitate the translation of evidence-based recommendations into routine clinical practice. A multilevel intervention model targeting patients, healthcare providers, clinical institutions, and healthcare policies has been proposed.^15^In a special report from the American College of Cardiology and the American Heart Association, a systematic review identified two particularly effective intervention strategies for improving the process of care and clinical outcomes: auditing clinical performance with feedback recommendations and conducting educational visits to healthcare providers.^15^

In conclusion, substantial evidence indicates that significant opportunities exist to improve LDL-c control among ASCVD patients in Brazil and globally, ultimately reducing the cardiovascular disease burden. Our collective failure to translate clinical evidence into action remains evident. Encouraging and supporting implementation research is essential to identify and promote the most effective strategies for integrating guideline recommendations into clinical practice.
